# Recent Advances in Green Hydrogen Production by Electrolyzing Water with Anion-Exchange Membrane

**DOI:** 10.34133/research.0677

**Published:** 2025-05-13

**Authors:** Lirong Zhang, Fang Qi, Rui Ren, Yulan Gu, Jiachen Gao, Yan Liang, Yafu Wang, Houen Zhu, Xiangyi Kong, Qingnuan Zhang, Jiangwei Zhang, Limin Wu

**Affiliations:** ^1^ College of Energy Material and Chemistry, Inner Mongolia Key Laboratory of Low Carbon Catalysis, Inner Mongolia University, Hohhot 010021, P. R. China.; ^2^ China Huadian Corporation Inner Mongolia Huadian Hydrogen Energy Technology Co. Ltd., Baotou 014500, P. R. China.; ^3^ Inner Mongolia Mengwei Hydrogen Energy Technology Co. Ltd., Hohhot 010021, P. R. China.; ^4^ Ordos Laboratory, Ordos 017000, P. R. China.; ^5^ Key Laboratory of Advanced Energy Materials Chemistry, Nankai University, Tianjin 300071, P. R. China.; ^6^ Department of Materials Science and State Key Laboratory of Molecular Engineering of Polymers, Fudan University, Shanghai 200433, China.

## Abstract

The development of clean and efficient renewable energy is of great strategic importance to realize green energy conversion and low-carbon growth. Hydrogen energy, as a renewable energy with “zero carbon emission”, can be efficiently converted into hydrogen energy and electric energy by electrolysis of water to hydrogen technology. Anion-exchange membrane water electrolysis (AEMWE), substantially advanced by nonprecious metal electrocatalysts, is among the most cost-effective and promising water electrolysis technologies, combining the advantages of proton exchange membranes with the proven technology of traditional alkaline water electrolysis and potentially eliminating the disadvantages of both. In this paper, the latest results of AEMWE research in recent years are summarized, including the AEMWE mechanism study and the hot issues of low-cost transition metal hydrogen evolution reaction and oxygen evolution reaction electrocatalyst design in recent years. The key factors affecting the performance of AEMWE are pointed out, and further challenges and opportunities encountered in large-scale industrialization are discussed. Finally, this review provides strong guidance for advancing AEMWE.

## Introduction

**Fig. 1.**
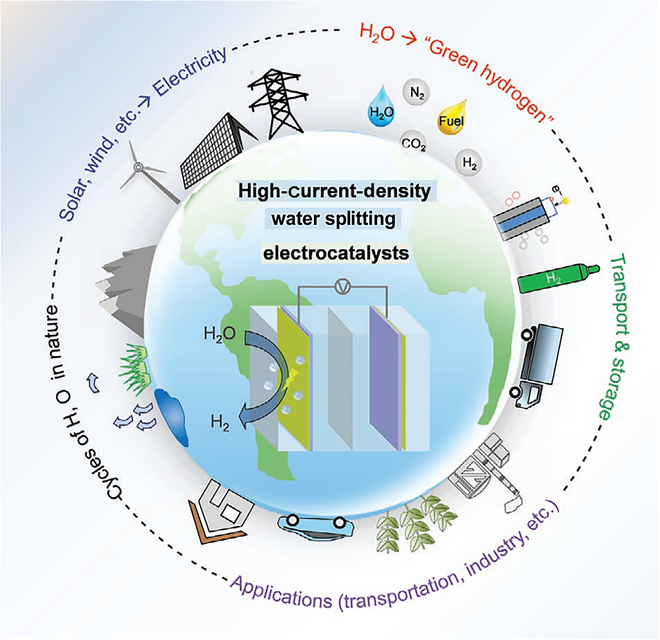
Schematic of “green hydrogen” [11].

As the global energy structure is transforming and environmental problems are becoming more and more serious, the importance of hydrogen energy as a clean and efficient energy carrier is becoming more and more prominent. Hydrogen energy can be converted into electricity by fuel cell technology and is widely applied in many areas such as power, chemicals, and transportation [1–4]. Hydrogen energy is considered to have an irreplaceable role in promoting the optimization of the energy structure. Hydrogen production from electrolyzed water is favored among many hydrogen production technologies for its high purity and nonpolluting characteristics [2]. Hydrogen production through the electrolysis of water is a green and sustainable method of hydrogen production [5]. Utilizing clean energy to produce H_2_ by electrolysis of water can effectively solve the problem of intermittency and instability of energy, and realize the efficient conversion and utilization of energy [4,6,7]. Furthermore, in applications such as zinc–air batteries, hydrogen is involved in the electrode reaction to increase energy density and efficiency while reducing environmental pollution, and is an important pillar in promoting the development of green energy and realizing the goal of carbon neutrality [8]. Hydrogen production technologies are generally categorized as “gray hydrogen”, “blue hydrogen”, and “green hydrogen” based on their carbon emissions. “Gray hydrogen” is hydrogen production from fossil fuels, with high carbon emissions; “blue hydrogen” is hydrogen production supplemented by carbon dioxide capture; “green hydrogen” utilizes renewable energy to generate electricity and then electrolyzes water to produce hydrogen. Green hydrogen utilizes renewable energy sources like solar and wind to produce electricity and then electrolyzes the water to produce hydrogen, which truly achieves zero carbon emissions [9]. Green hydrogen is the future of the energy industry and is produced through a process of electrolysis, in which renewable water and electricity are combined to form H_2_ and O_2_ under the influence of electricity. The hydrogen produced by this technology is extremely pure, up to 99.8%, with no carbon emissions and easy access to raw materials [10]. At the same time, an in-depth investigation of the basic principles of the electrolysis reaction is also a key link, which will optimize the existing energy structure and promote the sustainable development and wide application of green hydrogen (Fig. 1) [11]. Combining renewable energy with water electrolysis offers significant benefits, as excess electricity is stored chemically in the form of H_2_. In addition, the generated hydrogen and oxygen are directly used as primary energy sources in the fields of transportation and industry. Hydrogen can be used not only as a clean energy source but also in combination with fossil feedstocks to produce ammonia and synthetic fuels [1,12–14]. In addition, H_2_ can be combined with CO_2_ to synthesize green methanol, used as a synthetic fuel for transportation and other applications. These applications expand the scenarios in which hydrogen energy can be used and also provide important support for realizing the energy transition and sustainable development [15].

Water electrolysis is a process that separates water into H_2_ and O_2_ using an electrochemical reaction The theoretical minimum voltage required for water decomposition is 1.23 V at 101,325 Pa, 25 °C. [16,17]. This theoretical value is constrained by the high reduction potential of the anodic oxygen precipitation reaction [5,14]. To speed up the reaction process, it is necessary to apply an additional overpotential so that the cell voltage usually needs to be 2 to 3 V or higher to overcome the ohmic resistance. The cell voltage consists of 4 components: reversible overpotential, ohmic overpotential, activation overpotential, and concentration difference overpotential [18,19].

Since the 18th century, hydrogen production by water electrolysis technology has been developing continuously. According to the different electrolytes and operating conditions, 4 types of water electrolysis technology have been developed: alkaline water electrolysis technology (AWE), solid oxide electrolysis cell technology (SOEC), proton exchange membrane water electrolysis (PEMWE) technology, and AEMWE technology [6,20,21]. AEM electrolyzers operate in alkaline environments with nonprecious metal catalysts and cheaper metal bipolar plates while enabling differential pressure operation and higher current densities than conventional alkaline electrolyzers (LAs). Due to the inherent alkalinity of the AEM, pure water can be fed into the electrolyzer instead of an alkaline electrolyte, although current technology performs better and is more durable when fed with an alkaline electrolyte. The fact that AEM electrolyzers can use inexpensive, nonprecious metal catalysts will result in a significant reduction in the system cost of AEM hydrogen production, promising lower equipment and operating costs compared to traditional PEM and alkaline electrolyzer technologies. The ability to run at higher current densities means that more hydrogen can be produced with the same power input. This efficient energy conversion has a beneficial effect on reducing hydrogen production costs and improving the overall energy efficiency [22,23].

Due to their fast response characteristics, AEM electrolyzers can be started and stopped quickly, which makes them more suitable for applications with fluctuating renewable power. A more comprehensive techno-economic analysis is currently underway to estimate potential manufacturing system costs for large-scale production of AEM electrolyzers. Preliminary results indicate that the cost of AEM electrolyzers may be approximately 200 $/KW at relatively low production rates, and may ultimately be lower once higher production rates are reached. This low-cost potential is largely attributed to the lower material costs in the AEM electrolyzer design. AEMWE was first proposed in 2011 [24], and Zhuang’s group [25] realized the first application of pure water electrolysis with AEM in 2012. An electrolyzer was assembled with Ni–Fe anode and Ni–Mo cathode as catalytic electrodes and pure water as electrolyte, the electrolyzer was assembled with homemade AEM, and tests showed excellent performance similar to mature alkaline water electrolyzers. The AEM electrolyzer still requires significant development to improve stability and performance while keeping costs low. Due to lower material costs, the system cost of AEM electrolyzers may ultimately be lower than that of PEM electrolyzers, assuming that similar efficiency and durability goals are met. The commercial development of AEM electrolyzers may benefit from the system designs (including balance of plant) and large-scale fabrication techniques developed for PEM and LA electrolyzers. AEMWE technology, which has both the low-cost advantage of AWE and the high dynamic response advantage of PEMWE, has attracted extensive attention from the industry over the past few years and is expected to become the most promising technology, although it is still in the early stage of industrialization.

AWE technology is mature, highly industrialized, and relatively low cost due to the absence of precious metals in the cathode and anode electrode plates, but the low catalytic activity of the electrodes results in lower current density for maximum operation, lower efficiency, and gas crossover problems, leading to lower efficiency and safety hazards [26,27]. High-temperature SOEC technology is noted for its high efficiency (up to 90%) and environmental friendliness, but it requires additional energy to maintain high-temperature operation, which increases the overall energy consumption, and the durability and fabrication process needs to be improved [27,28]. PEMWE technology has developed rapidly in recent years and is now in the initial stage of commercialization. PEMWE technology uses a PEM as the solid electrolyte. Due to the low gas permeability of the polymer electrolyte membrane and the fast proton transport across the membrane in response to the input power, the PEM electrolyzer has the advantages of fast dynamic response and high purity of the hydrogen gas (usually around 99.99%). However, electrolyzers have a shorter lifespan, need to work in strongly acidic and highly oxidizing atmospheres, have higher costs for equipment, and rely on expensive materials such as iridium, platinum (Pt), and titanium [29–31]. AEMWE technology incorporates the reduced cost of an alkaline electrolyzer with the high efficiency of a PEM electrolyzer. AEM electrolyzer can reduce the dependence on precious metal catalysts; has good dynamic response characteristics, high stability, and purity; and can withstand higher current density during operation. In recent years, AEM technology has shown a broad development prospect due to its unique advantages and growing market scale; especially in the context of environmental protection and energy transition, the growth of AEM technology has received more and more attention and investment. Although it is still in the early stage of industrialization, the ionic conductivity of AEM is low, usually only about one-half of that of a proton exchange membrane. This is because OH^−^ ions conduct slower than H^+^ protons, and the structural design and material selection of AEMs limit the ion transport efficiency. The lower ionic conductivity increases the ohmic drop of the electrolyzer, leading to increased energy loss and reduced electrolysis efficiency, but as technology continues to innovate, AEM is crucial in driving the future’s energy transition. [32,33].

This paper summarizes the working principles, structural features, merits, and demerits of AEMs for hydrogen production in water, providing a basis for technology selection. Meanwhile, it focuses on the latest research and development of nonprecious metal oxygen evolution reaction (OER) and hydrogen evolution reaction (HER) catalysts for water electrolysis, especially on the development and structural innovation of new materials, such as Ni, Fe, Co, and other low-cost and abundant transition metal (TM) catalysts, which are aimed to improve catalytic efficiency and accelerate the technological progress using alloying and co-doping. This review also anticipates the evolving trends in AEM-based hydrogen production, from water electrolysis and the problems faced by industrial development, emphasizing its key role in promoting the achievement of the global carbon neutrality goal. Overall, this paper not only summarizes the current results but also provides a reference and guidance for the in-depth exploration and future development of hydrogen production from electrolytic AEMWE.

## AEMWE Hydrogen Production Technology

### Principles of AEMWE hydrogen production technology

**Fig. 2.**
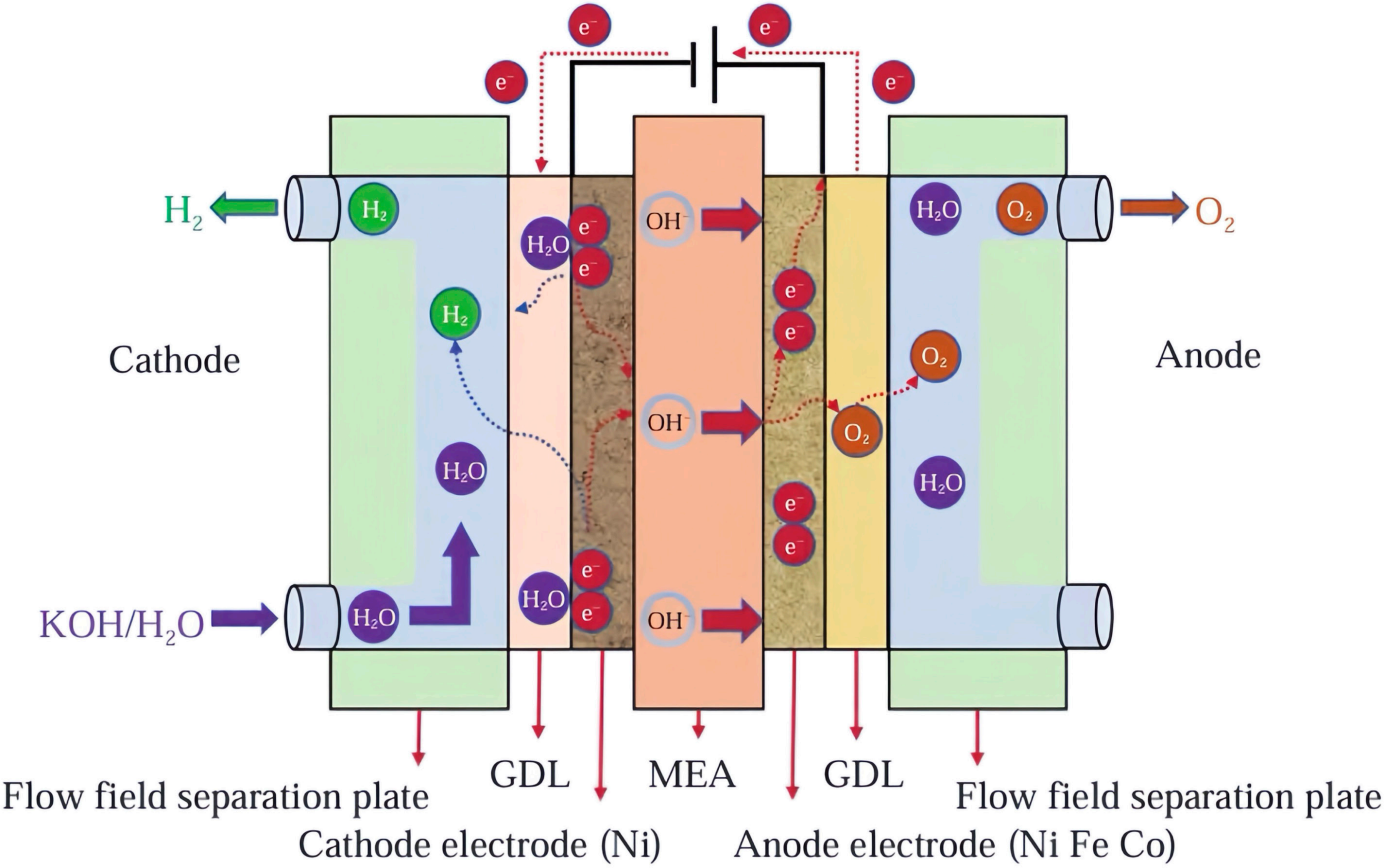
AEM electrolytic hydrogen production basic principles [164].

AEMWE is developed based on AWE and PEMWE technologies. Its structure is similar to the PEM electrolyzer, with AEM replacing PEM for OH^−^ transfer, as shown in Fig. 2. Pure water or alkaline solution is generally used as the electrolyte, and cost-effective nonprecious metal catalysts and AEM are used. Powered by 1.8 to 2.5 V, the devices electrochemically decompose water to produce O_2_ and H_2_ [34–37]. The alkaline water decomposition reaction of AEMWE and its thermodynamic potential are shown below:

$$\begin{array}{c} \begin{array}{c} \text{Cathode}:{\text{4H}}_{2}O\;+\;{\text{4e}}^{-}\to {\text{2H}}_{2}\;+\;{\text{4OH}}^{-}\;E_{0}=-0.828\;V\\ \text{Anode}:{\text{4OH}}^{-}\to O_{2}\;+\;{\text{2H}}_{2}O\;+\;{\text{4e}}^{-}\;E_{0}=0.401\;V\\ \text{Total reaction}:{\text{2H}}_{2}O\to {\text{2H}}_{2}\;+\;O_{2}\;E_{0}=1.23\;V \end{array} \end{array}$$

AEMWE requires few components such as electrodes, which can be made of nickel-plated stainless steel; it has a similar structure to the PEM electrode, enabling fast response and high current density while ensuring safe hydrogen production. AEMWE hydrogen production technology can effectively decompose water into H_2_ and O_2_ while maintaining high efficiency and safety [38].

### AEM structure characteristics and research progress

#### AEM electrolyzer assembly

The AEM electrolyzer is mainly composed of AEM, catalyst layer (CL), gas diffusion layer (GDL), and bipolar plate (BPP) in a sealed combination. The gas diffusion electrode (GDE) consists of CL and GDL, which is the core part of AEMWE. In GDE, GDL is responsible for the transportation of raw materials and products, and CL accelerates the water decomposition reaction to improve system efficiency. BPP is the transmission channel and needs to have good electrical conductivity and chemical stability. Graphite is usually used as the cathode of the BPP, while the anode of the BPP is in an oxygen-rich environment. To slow down the corrosion of the BPP, the use of expensive Ti plate is required [39,40]. Among them, the AEM diaphragm is the most important part of the AEM electrolyzer and directly determines the efficiency and operating life of the AEM electrolyzer. It can dramatically enhance electrode design and streamline the gas flow field. The AEM diaphragm separates the generated H_2_ and O_2_ gases while permitting the flow of OH^−^ and H_2_O [41–44]. The ideal high-efficiency AEM should have the following features: high mechanical strength, great thermal stability, superior chemical stability, high ionic conductivity, and the ability to effectively prevent electron and gas permeation. The main-chain structure of polymers is mainly concerned with providing both mechanical and thermal stability [45,46].

In recent years, researchers have significantly improved the performance of AEMs by introducing novel monomers and polymer structures such as poly aryl fluoroketones and polyaryl piperidines. These materials not only exhibit excellent durability in alkaline environments but also improve ionic conductivity by optimizing the microstructure of the membrane and ion transport channels [34,43]. For example, polyarylpiperidine-based AEM maintains good conductivity and dimensional stability after operating at 80 °C for more than 1,600 h [47]. In addition, these new AEMs have performed well in practical applications, such as achieving high power density and low voltage decay rates in fuel cells. Wang’s group [43] at Tsinghua University designed a class of heteroatom-free rigid molecules (HFMs), which were introduced into the polymer backbone to make AEM, which showed excellent chemical stability and high conductivity in alkaline environments.

#### Research progress of AEM materials

AEMs are prepared by various methods, mainly including interpenetrating polymerization, solution polymerization, in situ polymerization, electrostatic spinning, hole filling, and polymer blending. Among them, the polymer blending method is currently the mainstream preparation method, which has the advantages of good quality finished products, a rich variety of raw materials, and a simple preparation process. In addition, the microphase separation technology is also widely used in the preparation of AEMs. By introducing twisted units or constructing branched structures in polymers, the free volume in the polymers can be increased, which enhances the ion transport and reduces the solubility ratio of the membranes. For example, all-carbon backbone polymers synthesized based on ultra-strong acid-catalyzed reactions with high chemical stability have become ideal candidates for next-generation AEMs [48–51]. To optimize the performance of AEM, researchers have optimized it in several ways. First, the degradation of membranes in alkaline environments can be effectively reduced by introducing ion-exchange groups with high alkali stability, such as piperidine and imidazole. For example, the AEM based on piperidine cation showed excellent stability under alkaline conditions. Second, in terms of ionic conductivity, by adjusting the ion exchange capacity (IEC) and optimizing the microstructure of the membranes, high conductivity can be achieved while maintaining low swelling. Moreover, the mechanical strength and ionic conductivity of the membranes could be significantly improved by introducing nano-fillers or constructing organic/inorganic composites in the AEM [44,52].

## Research Progress of AEM Electrode Materials

The demand for catalysts in AEM electrolyzers focuses on high activity, high stability, low overpotential, cost-effectiveness, and good interfacial bonding. In particular, nonprecious metal catalysts are ideal for AEM electrolyzers owing to their inexpensive and abundant sources [53,54].

### Different catalysts/electrodes

**Fig. 3.**
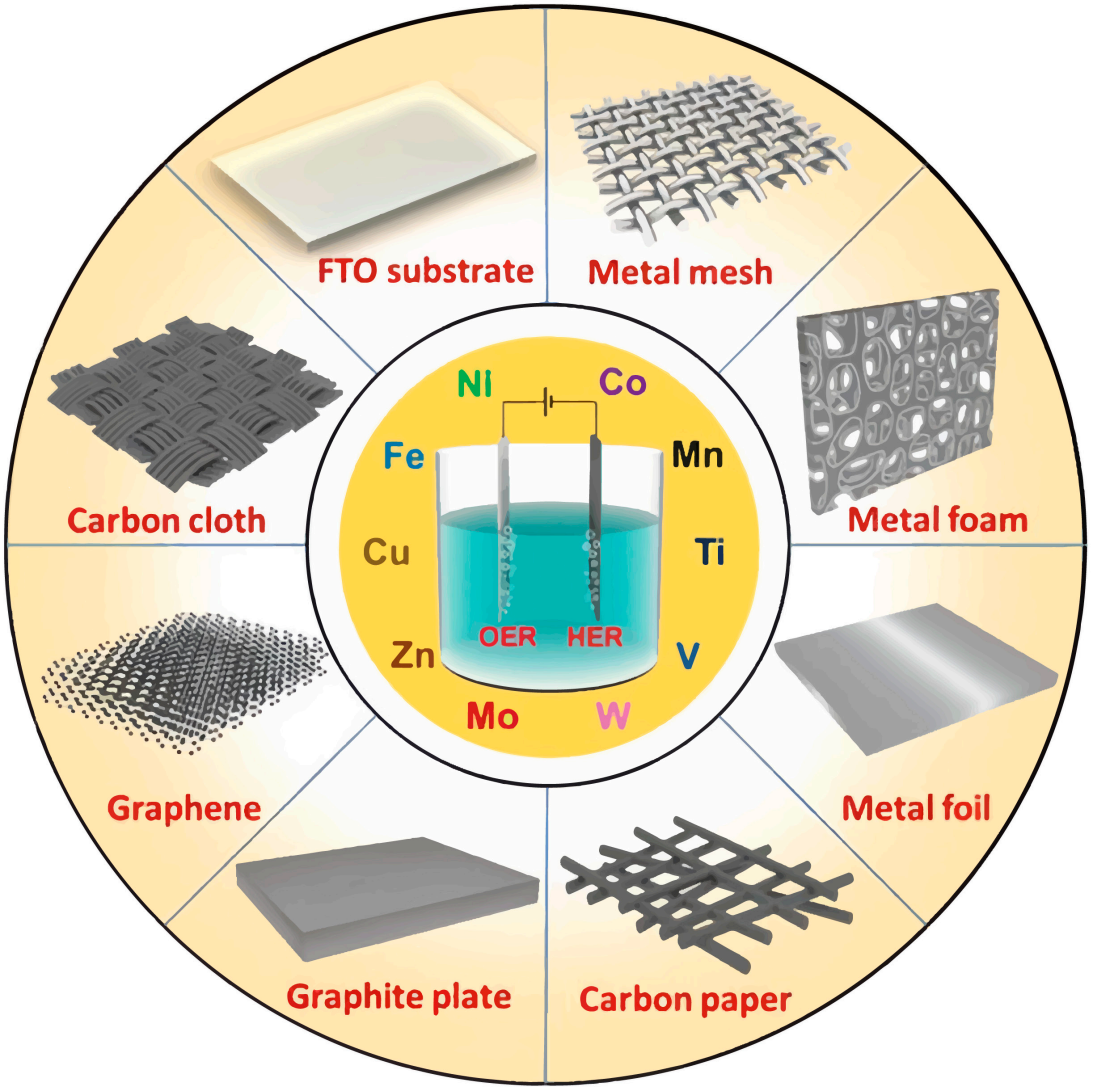
Substrate material diagram for self-supporting electrodes [165].

Current catalyst forms are mainly categorized into self-supported electrodes and powder catalysts. Self-supported electrodes are typically electrodes obtained by growing the active material in situ on a conductive substrate, which does not require the participation of adhesives to obtain catalytic materials that are tightly bonded to the base and do not block the active sites. Typical preparation methods include hydrothermal (solvothermal), electrodeposition, and vapor phase deposition. The main elements involved are TM elements [55–57]. Self-supporting electrodes are constructed in 2 main ways: (a) porous self-supporting conductive material itself as the skeleton, and load the active material on the surface of the skeleton, ensure excellent contact between the material and the substrate, and at the same time left behind a wealth of pores can realize the rapid transmission of liquid-phase ions to achieve high multiplicity performance. This skeleton material is generally optional carbon cloth, foam metal (nickel, copper, aluminum, etc.), pyrolyze porous materials (paper, sponge, cotton), and so on (Fig. 3) [56,58,59]. (b) Directly construct the electrochemically active material into a structure with a self-supporting function. Such self-supporting materials can be chosen from carbon fiber, carbon nanotubes, graphene, conductive polymers, etc. Powder-type catalysts combined with an anionic membrane and GDL can form membrane electrodes with different electrode configurations. This approach requires anionic ionomer (AEI), which involves the design of catalyst slurry and catalytic layer [60].

### OER catalysts for the oxygen precipitation reaction

**Fig. 4.**
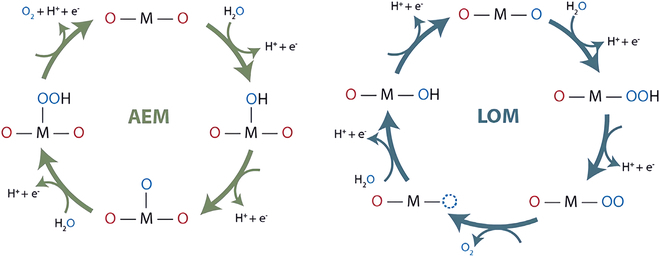
Mechanisms of the OER response [68].

OER as a half-reaction of water electrolysis requires a complex 4-electron transfer process. As shown in Fig. 4, it can be carried out by 2 different pathways: the adsorbate evolution mechanism (AEM) and the lattice oxygen oxidation mechanism (LOM), with the main difference being that oxygen molecules are formed in different ways [31,61–63]. The AEM mechanism is a concerted proton–electron transfer reaction centered on metal ions, and the metal undergoes a redox reaction. At each step, protons are transferred to the electrolyte, and the metal active sites continuously bind OH^−^ in the electrolyte to form M–OH, M–O, and M–OOH intermediates. The strength of adsorption of the intermediate on the active site decides the catalytic performance. Too high adsorption strength will lead to difficulties in the following reaction, and too weak adsorption strength will reduce the coverage of the reactants. Ideally, the adsorption strength of the intermediate must be at an adequate level [64–66]. Eventually, the M–OOH intermediate removes the proton and binds to OH^−^ to give the product oxygen [67,68].

The LOM mechanism is a nonsynergistic proton–electron transfer reaction centered on the oxygen site, and it is oxygen that undergoes the redox reaction. During the first steps, the same M–OH and M–O intermediates are generated, then the lattice oxygen in the neighboring metal position directly undergoes the electron transfer-free O–O radical coupling, and finally the electrons are removed to release oxygen [69].

### Precious metal OER electrode materials

IrO_2_ and RuO_2_ have proven to be the most advanced catalysts available with outstanding electrical conductivity and OER catalytic activity. Among them, IrO_2_ was used as an OER benchmark catalyst. The catalytic activity of amorphous IrO_2_ is higher, and crystalline/rutile phase IrO_2_ has stronger stability [70–72]. Precious metal-based catalysts are hardly applied to AEMWE due to their exorbitant cost, which has become a key constraint to its development. However, in AEMWE, the nonprecious metal catalysts have become a popular choice for oxygen precipitation reaction due to their high pH environment, which is milder compared to an acidic environment, with the advantages of low cost, abundant resources, and great activity [65,73,74].

### Nonprecious metal OER electrode materials

#### Metals/alloys

TM alloys exhibit efficient catalytic performance in OER reactions due to their excellent conductivity and synergistic effects between multi-metallic catalytic sites. NiFe alloys are an effective means of enhancing OER activity by promoting electron transfer and thus accelerating the OER reaction [75,76]. During the OER activation process, the alloy catalyst obtained by electrodeposition undergoes an in situ remodeling phenomenon to form NiFe hydroxide on the CL surface. This reconfigured NiFe hydroxide acts synergistically with the deposited NiFe alloy to make the catalyst catalytically active. Therefore, in the actual OER reaction, the TM alloy catalyst may mainly act as a “pre-catalyst”, while the final metal (oxygen) hydroxide is the real catalytically active and stable phase of OER under an alkaline environment [77].

**Fig. 5.**
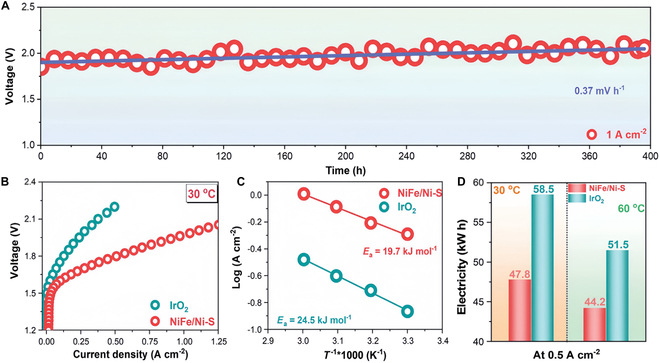
Comparative performance of AEMWEs. (A) Polarization curves. (B) Electrochemical impedance spectroscopy (EIS) at 1.7 V. (C) Tafel plots. (D) AEMWE long-term durability recorded at 60 °C, 1.0 A cm^−2^ constant current condition [78].

Chen et al. [78] have synthesized an anodic catalyst to grow Ni_3_S_2_ in situ on nickel–iron alloy nanoparticles and developed a sulfur source limitation strategy. The results showed a superpotential of 239 mV for 10 mA cm^−2^. The AEMWE anode material NiFe/Ni–S had a cell voltage of 1.664 V at 500 mA cm^−2^ and was able to maintain it for 400 h at 1.0 A cm^−2^. The findings of this research offered a new idea for fabricating nonprecious metal powder electrocatalysts, which are expected to be applied in the future AEMWE technology (Fig. 5).

#### Oxidizing compounds

TM oxides, particularly spinel oxides, are extensively utilized in electrolytic water hydrogen production owing to their excellent catalytic performance and stability. Through doping and defect modulation, their morphology and structure can be effectively optimized. With abundant redox active sites and good chemical stability, TM oxides are more widely studied nonprecious metal OER electrode materials. Their structures are diverse, and their catalytic performance can be optimal by adjusting the composition, valence, and crystal structure of metal elements [79–82]. However, their electrical conductivity is usually poor, thus limiting the overall catalytic performance.

**Fig. 6.**
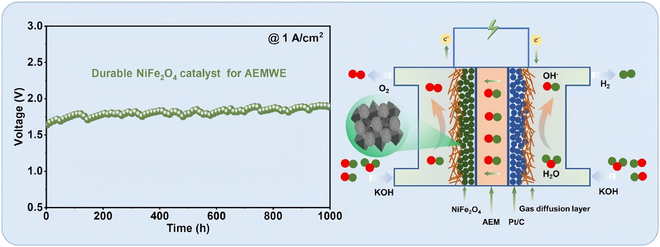
AEM electrolyzer performance of NiFe_2_O_4_ [85].

Manganese oxide with nano-display structure was grown in situ on titanium foil by electrodeposition under alkaline conditions. The surface area is very large at 538 mV overpotential, and the redox onset potential is 1.546 V [83]. Yoon et al. [84] prepared a Ti-doped NiFe_2_O_4_ (Ti-NFO) nanosheet catalyst by thermal calcination. It has been found that the introduction of Ti improves the intrinsic activity of Ti-NFO. In the KOH electrolyte, the electrode required only 230-mV overpotential to attain 10 mA cm^−2^. When Ti-NFO is used in AEMWE, Ti-NFO can reach 1 A cm^−2^ at 1.73 V and can be operated stably at 500 mA cm^−2^ for 500 h. Wang et al. [85] synthesized a polymerized polymer with the strategy of combining hydrothermal and thermal reduction reactions. The NiFe_2_O_4_ catalyst was assembled into an AEMWE anode catalyst (Fig. 6) that required only 1.64 V to reach 1 A cm^−2^ and was maintained for 1,000 h.

#### Hydroxide compounds

TM layered double hydroxides have recently attracted a great deal of attention as catalysts and have been studied to date as follows [86–88]. Layered double hydroxide catalysts (LDHs) are 2-dimensional (2D) anionic layered compounds with a hydromagnesite-like body layer of interlayer anions consisting of a positive charge and a charge balance [89–92]. The structure of LDH consists of monolayer or multilayer nanosheets, thus displaying more active sites. TM hydroxides are widely used as oxygen precipitation reaction catalysts in AEMWE due to their excellent catalytic activity and unique electron distribution. These materials have great alkaline stability and can also modulate their catalytic performance through the flexibility of oxygen vacancies and unique electron distribution [31,93–95].

In general, the LOM pathway provides higher catalyst OER activity than the AEM pathway because it bypasses the O–O bonding. Given the multivalent nature of cations and the outstanding ability of TM spinel oxides to switch between different oxidation states, efforts have been made to dope spinel oxides with heteroatoms and substituted ions to modulate the OER performance via the favorable LOM pathway [61]. Effective strategies need to be found to regulate the lattice oxygen redox of spinel oxides and bypass the limitations of the AEM scalar relationship [96].

**Fig. 7.**
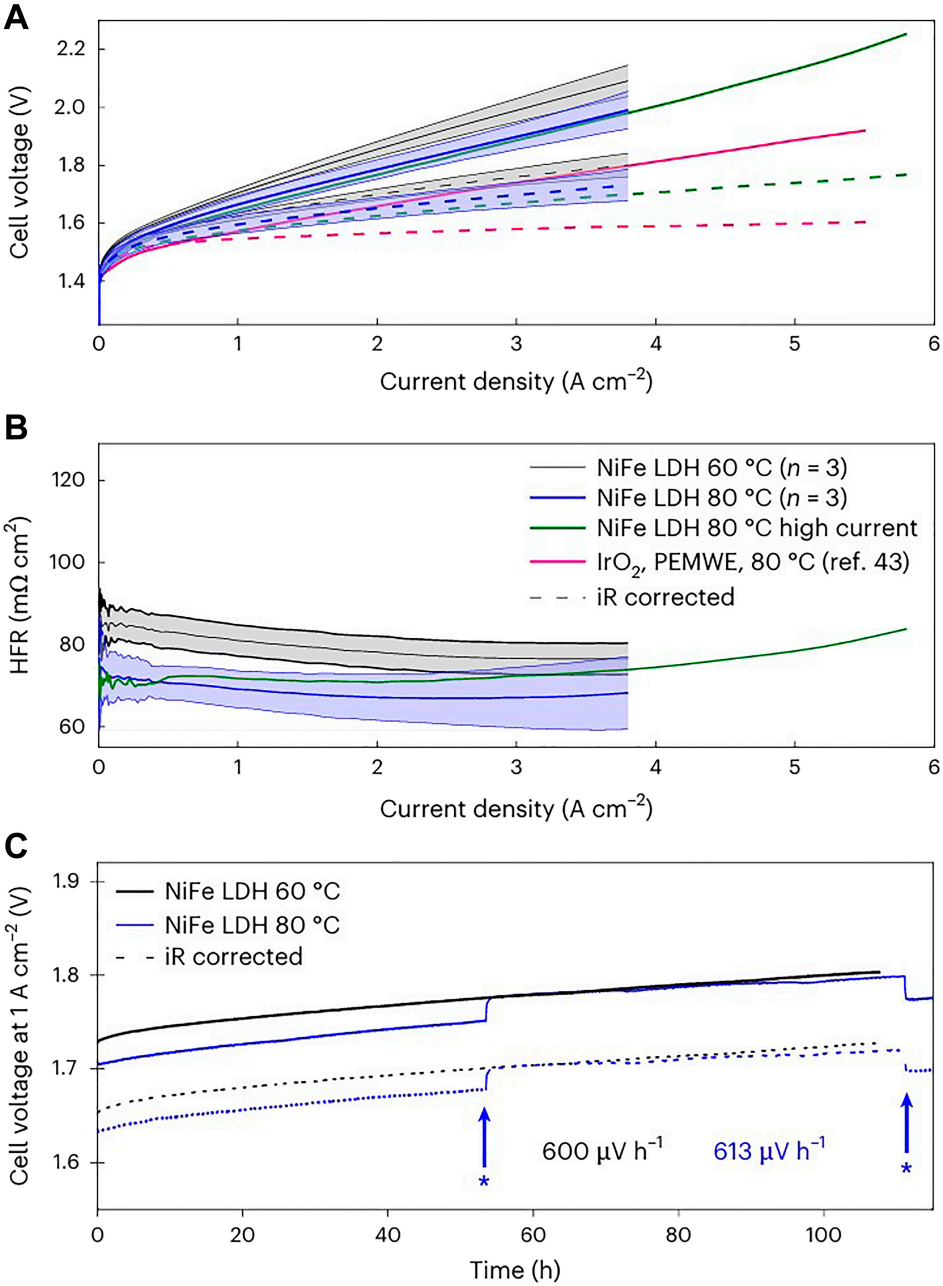
(A) AEMWE cell polarization curves for NiFe-LDH and Pt measured in 1 M KOH. (B) Plot of HFR values versus current density. (C) AEMWE performance: 53 h with an asterisk denotes the point at which the cell temperature became 70 °C; 110 h with an asterisk denotes when the temperature rose to 80 °C again [97].

LDHs based on Ni and TMs Fe, Co, and Mn were proposed and systematically evaluated by Klingenhof et al. [97] to successfully replace conventional as well as catalysts. Nickel and its alloys are low-cost and catalytically active, and their reactivity and stability are further enhanced by the optimized design of TMs. The LDH catalyst not only promoted the oxygen production reaction of electrolyzed water but also further improved the catalytic performance. The electrochemical performance of the AEMWE system, particularly its polarization behavior at high current densities and long-term stability, was evaluated. Single-cell measurements at 60 and 80 °C demonstrate the excellent performance of the NiFe LDH catalysts in AEMWE (Fig. 7). Compared with the PEMWE system, the AEMWE single cell exhibited lower resistance loss and higher electrolysis efficiency with the NiFe LDH catalyst, indicating that this catalyst has a promising application and commercialization potential.

**Fig. 8.**
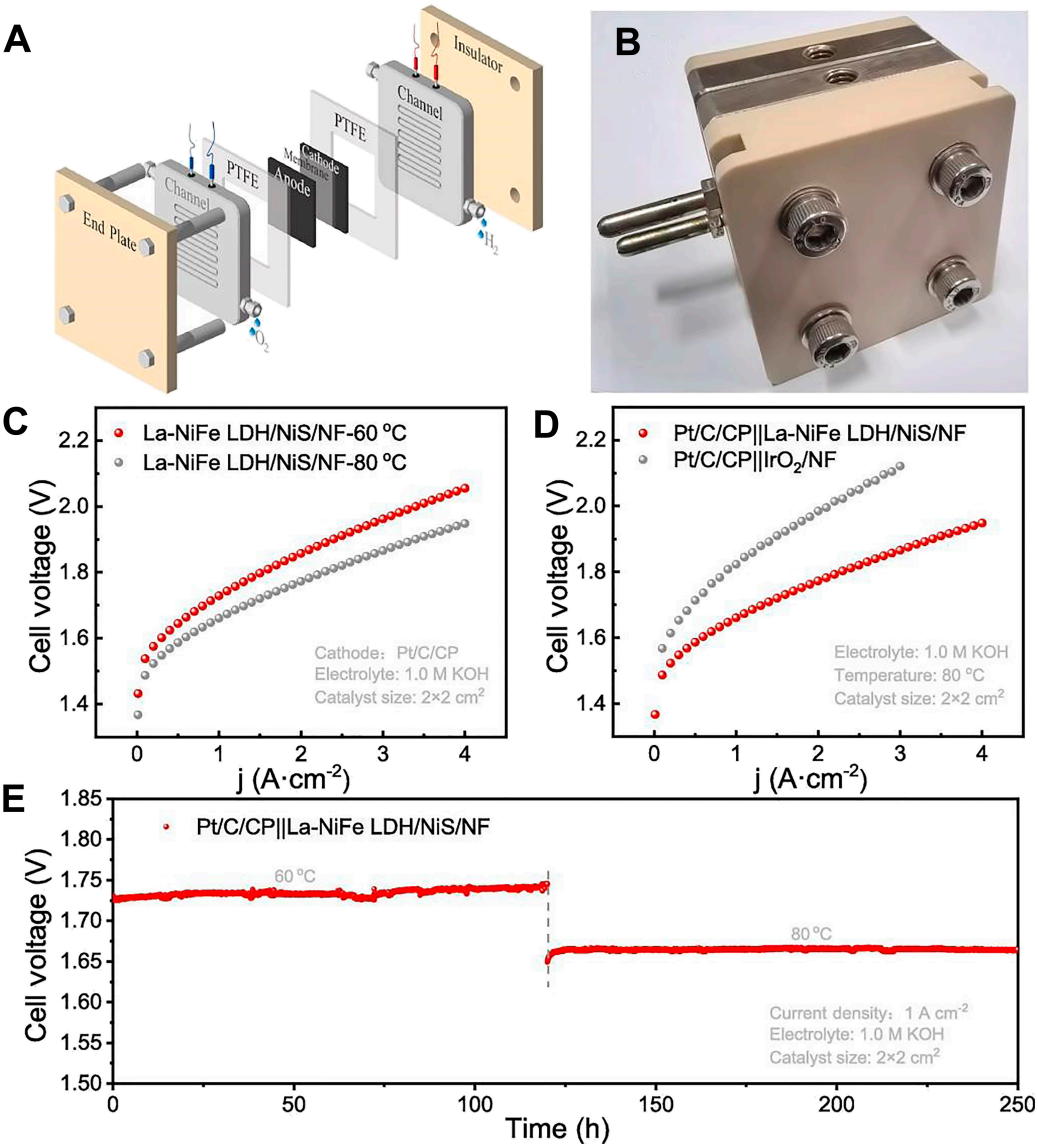
(A) Schematic of the structure of the AEMWE cell. (B) Performance of AEMWE at different temperatures (C) and 80 °C. (D) Performance of AEMWE. (E) Long-term durability of the material at 1 A cm^−2^ [100].

Nowadays, scientists have introduced rare earth elements into LDH through various methodological strategies to improve the stability and conductivity of LDH. These measurements not only improved the activity and durability of OER but also optimized the electronic structure and ion migration paths of the materials, providing new ideas and approaches [98,99]. Song et al. [100] directly etched Ni foam substrates for vulcanization. This process enhanced the conductivity of the substrate and formed a nanosheet layer rich in NiFe-LDH. As a result, the intrinsic activity was boosted, and more active sites were exposed due to the increased junction nanolaminates. A single-step La ion doping electrodeposition process was applied to NiFe LDH, effectively regulating electron transfer within the NiFe LDH/NiS heterojunctions and lowering the OER reaction’s activation energy. When using La-NiFe LDH/NiS/NF as the anode, the water electrolysis system exhibits long-term durability and activity compared to devices using IrO₂ as the anode or most AEM-based water electrolyzers. This compound meets the stringent requirements for catalytic activity and stability under large current conditions. Rare earth element doping further promotes electron transfer and optimizes the binding strength and reaction activation energy of oxygen intermediates. Its performance at 1 A cm^−2^ @ 80 °C with 1.66 V exceeds the most advanced (Fig. 8). This provides a precious guideline for the development of next-generation AEMWE technologies.

#### TM phosphides/sulfides

**Fig. 9.**
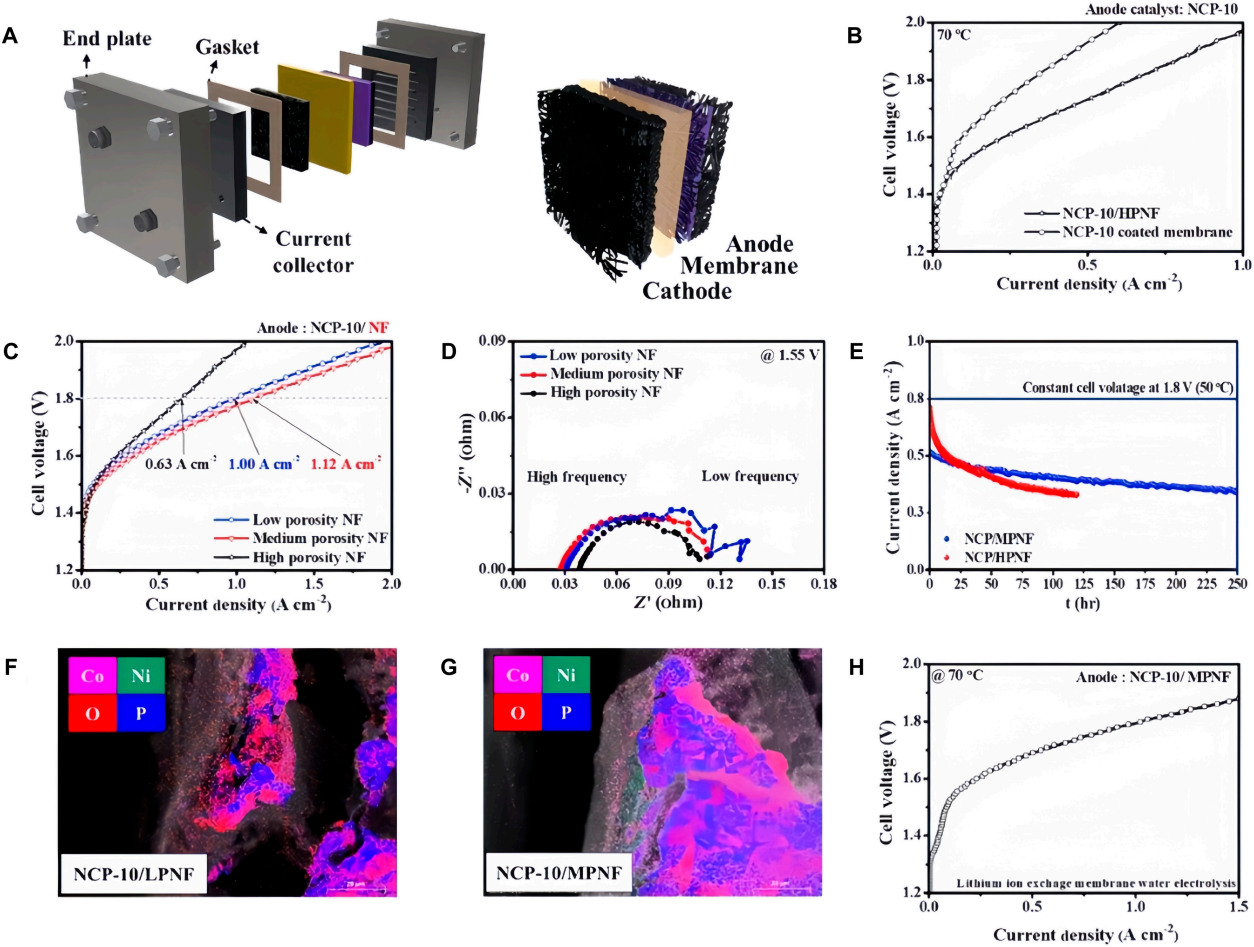
(A) Diagram of AEMWE device. (B) Polarization curve of NCP-10. (C) Polarization curve. (D) Nyquist plot. (E) Long-term stability testing. (F) Stability test. (G) Scanning electron microscopy (SEM) image. (H) Polarization curves of single cell [105].

TM phosphides (TMPs) are considered to be an important class of catalytic materials for OER. TMPs exhibit excellent electrical conductivity, facilitating efficient electron transfer during electrocatalytic processes and enhancing catalytic performance. Additionally, their surfaces readily generate active sites, which help lower the overpotential for the OER [101–104]. However, there are some drawbacks. Its long-term stability in strong alkaline is poor, and it is prone to surface oxidation or corrosion, leading to a decline in catalytic performance. Park et al. [105] introduced spherical Ni–CoP as an efficient OER anode. They reported an OER catalyst spherically doped with Ni–CoP, which may increase the energy density of the atomic energy is CoP Fermi energy level upon the addition of Ni, and can improve the charge transfer efficiency. The electrocatalytic activity of AEMWE was found to be dependent on the porosity as a catalyst substrate. NCP-10 used as an AEMWE catalyst provides 1.12 A cm^−2^ at 1.8 V for 250 h (Fig. 9).

**Fig. 10.**
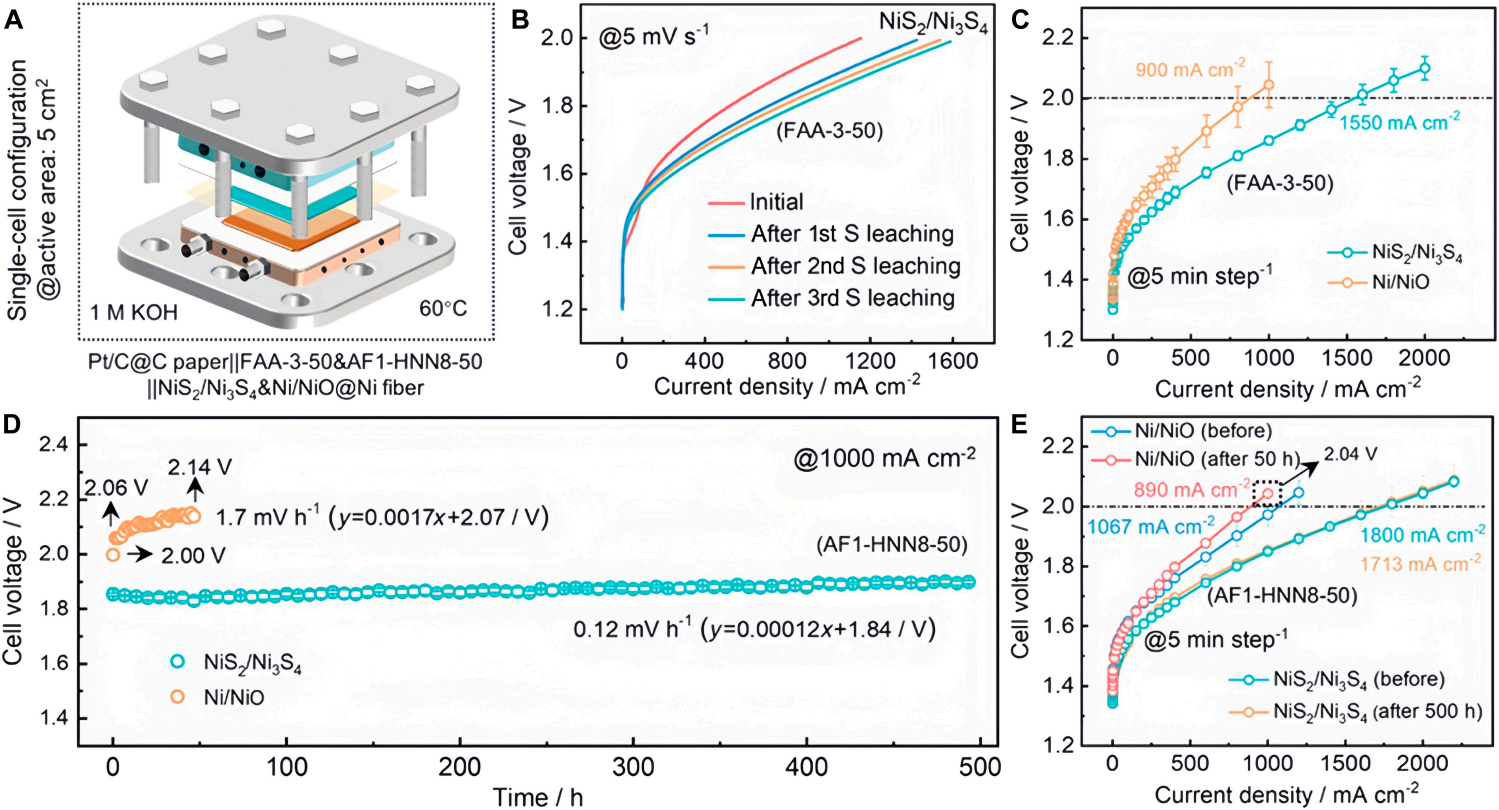
(A) Schematic diagram of the single-cell structure. (B) Polarization curves of Pt/C||FAA-3-50||NiS_2_/Ni_3_S_4_ cell at 5 mV^−1^. (C) Polarization curves after conditioning at 1.7 V for 6 h by a galvanostatic method (5 min step^−1^). (D) Stability at 1,000 mA cm^−2^. (E) Polarization curves before and after stability test [112].

TM sulfides, abundant in active sites, efficiently enhance electrocatalytic reactions. Their catalytic performance can be further improved by modifying composition, morphology, and crystal structure, making them highly advantageous as OER catalysts [106,107]. Modulating the electronic structure of TM sulfides improves OER efficiency in alkaline media [108–111]. Xia et al. [112] designed a nickel-rich NiS*_x_*/Ni(OH)_2_/NiOOH with 374 mV at 50 mA cm^−2^ and a stable voltage increase of only 0.1% after 65 h of testing at 10 mA cm^−2^. When integrated into an electrolyzer, the system reached 1,800 mA cm^−2^ at 2.0 V (Fig. 10).

#### Metal-organic frameworks and polyoxometalates

**Fig. 11.**
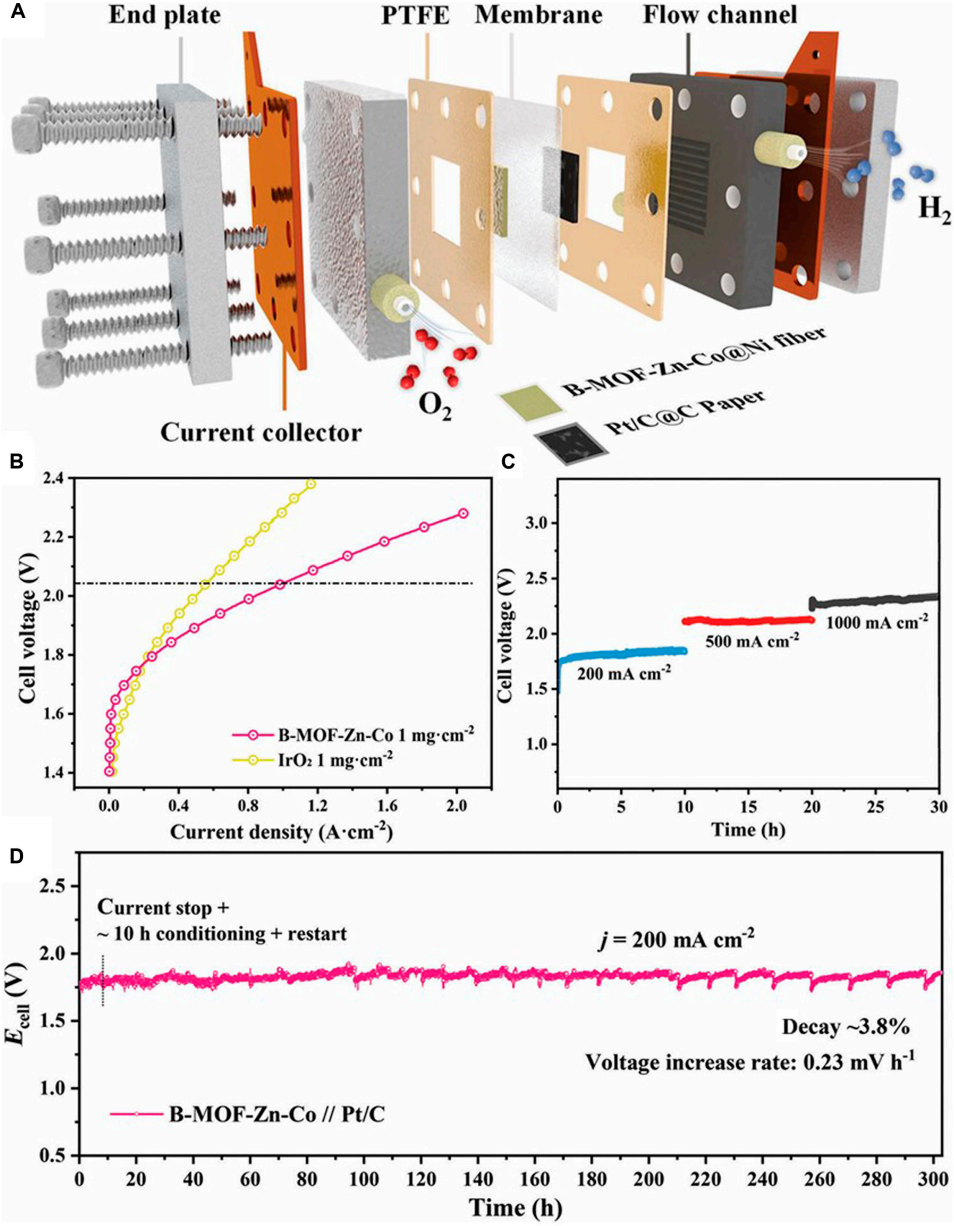
(A) Schematic of AEMWE. (B) Current-density versus voltage (*I*–*V* ) curves of AEMWE cell. (C) Timing test at different current densities. (D) Stability test in the AEMWE electrolyzer [117].

Metal-organic framework (MOF) materials have highly ordered porous structures and designable chemical compositions, which can be converted into metal oxides, carbon materials, or metal–carbon/nitrogen composites with abundant microporous structures and active sites by pyrolysis, etching, etc., and these derivatives exhibit good catalytic performance in OER reactions. However, some MOF materials are not stable enough under strong alkaline or high-temperature environments and are prone to structural collapse [113–116]. Lin et al. [117] synthesized B-MOF-Zn-Co material by solvothermal method, which has 362 mV at 100 mA cm^−2^ under alkaline conditions. During electrolysis, the compound is completely converted to B-doped CoOOH, which is a practically active material. When integrated into an AEMWE cell, B-MOF-Zn-Co enables stable and continuous operation for over 300 h at 200 mA cm^−2^ (Fig. 11). This study presents an in situ electrochemical reconstruction approach to develop stable and efficient OER catalysts for AEMWE.

Polyoxometalates (POMs) have gained attention as a promising and attractive option. POMs are composed of clusters of inorganic redox-active TM oxide ions (such as those containing V, W, and Mo) that are interconnected by oxygen atoms. These structures possess numerous redox centers, which make them particularly effective in multi-step electron transfer reactions [118,119]. They have an organized 3D skeletal structure that can be easily adjusted. POMs have shown significant advantages and features in AEMWE anodic OER, which can optimize the catalyst structure and significantly improve the catalytic activity by the wet etching method. The POM-treated catalysts exhibit low overpotential and high durability in alkaline electrolytes, and their high ionic conductivity and reversible oxidation–reduction activity make them very promising for practical applications. Reduction activity makes it a great potential for practical applications and provides an important material basis for an efficient and stable AEMWE system [120–122].

**Fig. 12.**
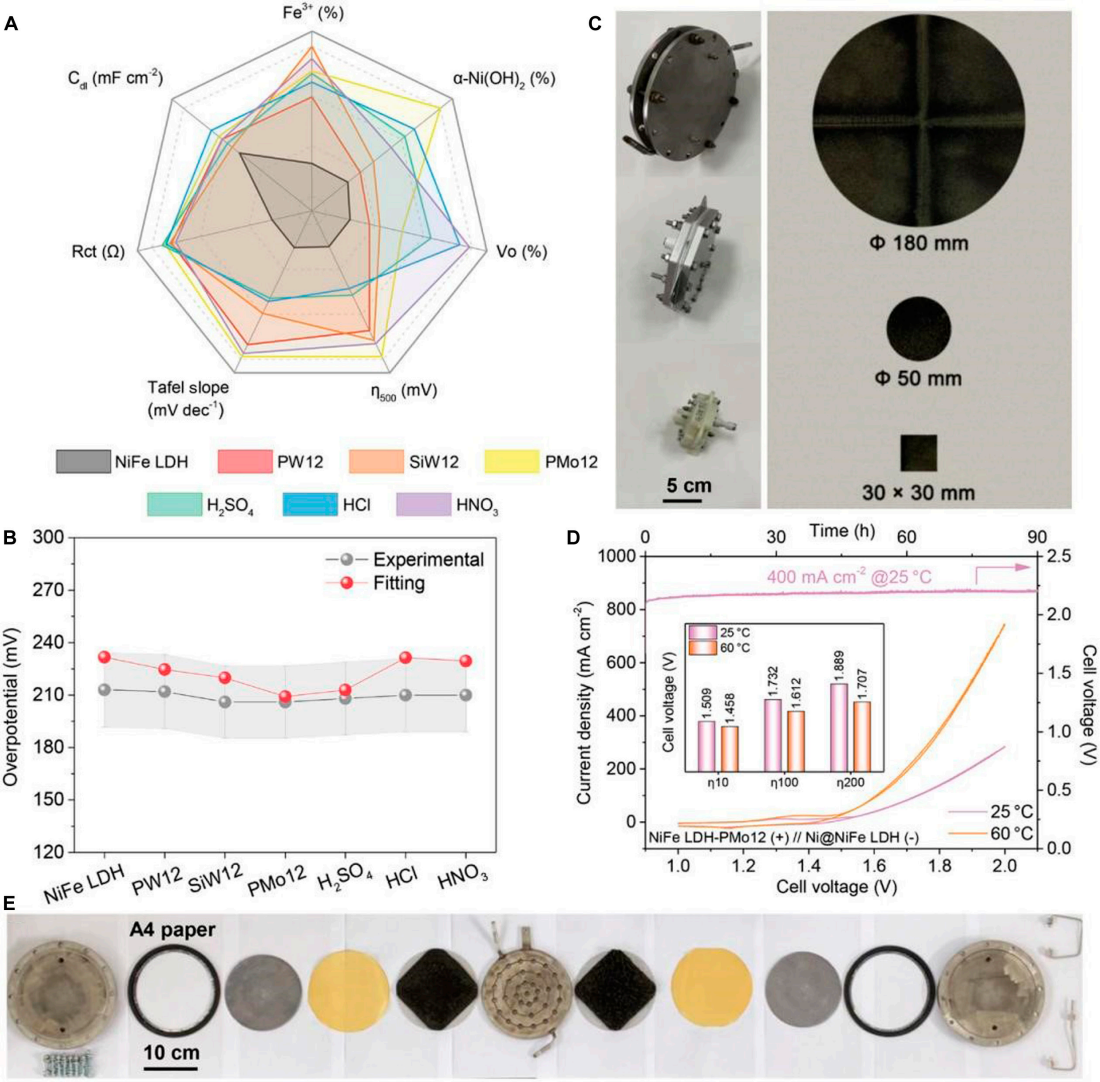
(A) Comparison of NiFe LDH performance determinants. (B) Comparison of electrocatalytic performance. (C) Schematic diagram of the electrolysis cell. (D) Polarization curves of overall water splitting measurement and the stability test in AEM cells for 90 h. (E) Stack structure of AEM electrolysis cell for operation [123].

Cai et al. [123] reported NiFe-LDH prepared by an electrodeposition strategy and reconfigured by a POM etching method, which significantly improved its OER performance. The POM etching method, which can skillfully reconstruct NiFe LDH, includes 3D morphology nanocropping, Fe^3+^ and *α*-Ni(OH)_2_ reactive species reconfiguration, and embedding of POM polyanion clusters. Electrocatalysts with diameters up to 180 mm were successfully developed at a low cost and high performance. These advancements highlight the viability and superiority of the NiFe LDH-PMo12(+)||Ni@NiFe LDH(−) system for use in AEM electrode stack batteries. The improved AEMWE based on NiFe LDH has achieved more than 300 h of stable operation at 200 mA cm^−2^ (Fig. 12).

### HER electrode materials

**Fig. 13.**
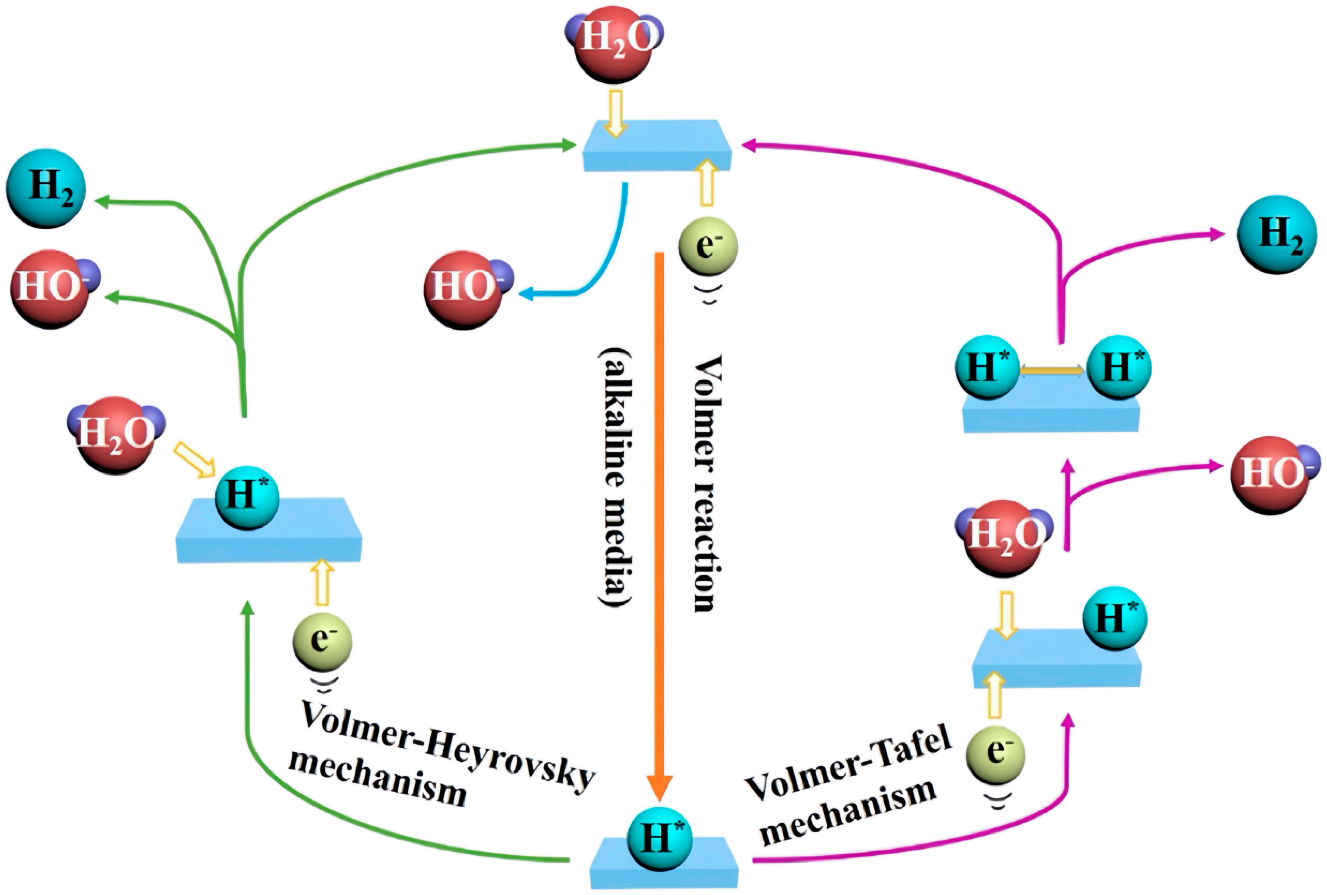
Schematic diagram of HER reaction mechanism [125].

HER is a key half-reaction that occurs at the cathode during water electrolysis and consists of the following steps: the Volmer step, the Heyrovsky step, and the Tafel step (Fig. 13) [124–127]. In alkaline media, water molecules act as a proton source to split hydrogen atoms and adsorb them onto the metal active sites. Subsequently, the adsorbed hydrogen atoms form hydrogen molecules in the Heyrovsky or Tafel step. In the Heyrovsky step, the adsorbed hydrogen atoms combine with water molecules and electrons in the electrolyte to form hydrogen. In the Tafel step, 2 neighboring MH* combine directly on the catalyst surface to form hydrogen [128,129].

#### Precious metal catalysts

The application of precious metals in HER has always been a research hotspot, especially Pt and its alloys, which are considered to be the best-performing HER catalysts at present due to their low overpotentials, high exchange current densities, and excellent reaction stability. However, the widespread use of precious metals is hindered by their high cost [130–133]. In particular, the development of single-atom catalysts (SACs) has provided new ideas to improve the utilization efficiency and catalytic performance of precious metals. SACs are single-metal atoms scattered on a carrier to form highly dispersed catalytic active sites. This structure not only greatly improves the utilization of precious metals but also exhibits excellent catalytic performance because of the unique properties of individual atoms. For example, the noble metal is loaded in the form of individual atoms on a suitable carrier and the highly active edge sites of the catalyst particles are fully exposed [134–137].

Lim et al. [137] have developed an efficient electrocatalyst for AEMWE that exhibits high kinetic performance in the alkaline HER. By using Ni nanoparticles as artificial monolithic sites adjacent to Pt single atoms, the research team designed an advanced Pt single-atom-based electrocatalyst, which showed better performance than the Pt/C commercial catalyst in the system of AEMWE at high current densities.

#### Nonprecious metal catalysts

##### TM-based catalysts

TMs and their compounds are rich in d-electronic structures, which are capable of effective adsorption and desorption with hydrogen atoms, thus promoting the HER reaction. The catalytic properties can be optimized by modulating the composition, valence, and nanostructure of the metals, which exhibit good HER activity in alkaline electrolytes [138–144]. TM nitrides have a distinctive structure and therefore excel in electrocatalysis with catalytic properties similar to those of noble metals [145]. Similarly, TM sulfides and phosphides are advantageous for HER, with sulfides offering high activity, abundant active sites, and tunable electronic properties, while phosphides provide excellent conductivity, stability, and Pt-like performance. Both are cost-effective and resource-rich, making them ideal for large-scale applications. However, these materials still have an almost unlimited scope for performance in large-scale applications.

**Fig. 14.**
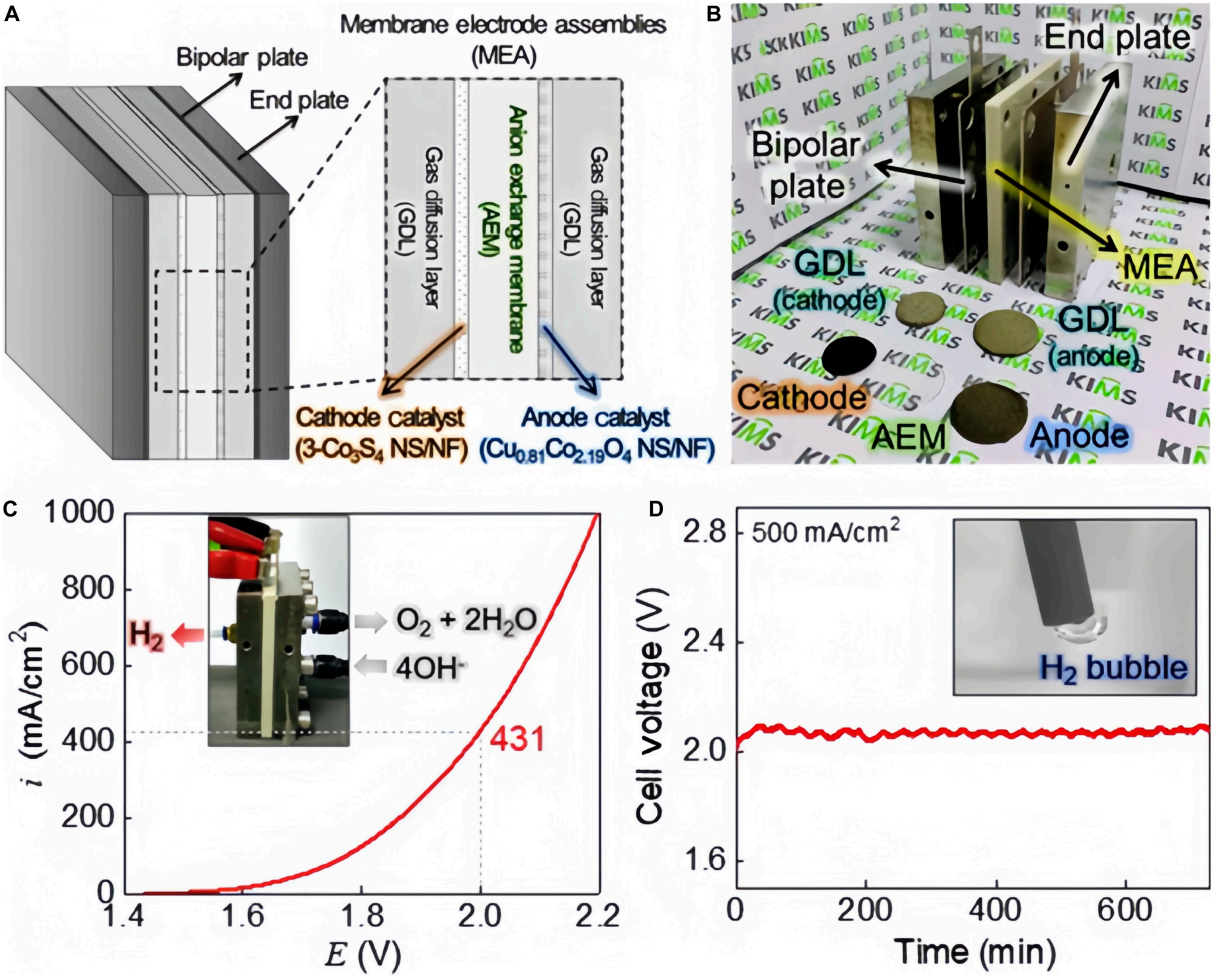
(A) Schematic diagram of AEMWE. (B) Single-cell AEMWE. Application of Co_3_S_4_ and Cu_0.81_Co_2.19_O_4_ NS/NF to HER and OER electrodes. (C) Linear sweep voltammetry (LSV) polarization curves. (D) Durability test [146].

Park et al. [146] Co₃S₄ NS/NF prepared by electrodeposition and sulfidation is an efficient HER electrocatalyst for alkaline water electrolysis and single-cell AEMWE systems. This catalyst has a vertically or tilted-grown 3D structure that provides numerous active sites, and the successful conversion from Co₃O₄ to Co₃S₄ was achieved during the sulfidation process, which significantly improved its electrocatalytic performance. The Co₃S₄ NS/NF electrocatalyst showed the lowest electrocatalytic activity at −10 mA/cm^2^ and showed the lowest overpotential of 93 mV, which was excellent in N₂-purified 1 M KOH solution. In addition, a single-cell AEMWE showed 431 mA/cm^2^ with a cell voltage of 2.0 V (Fig. 14).

**Fig. 15.**
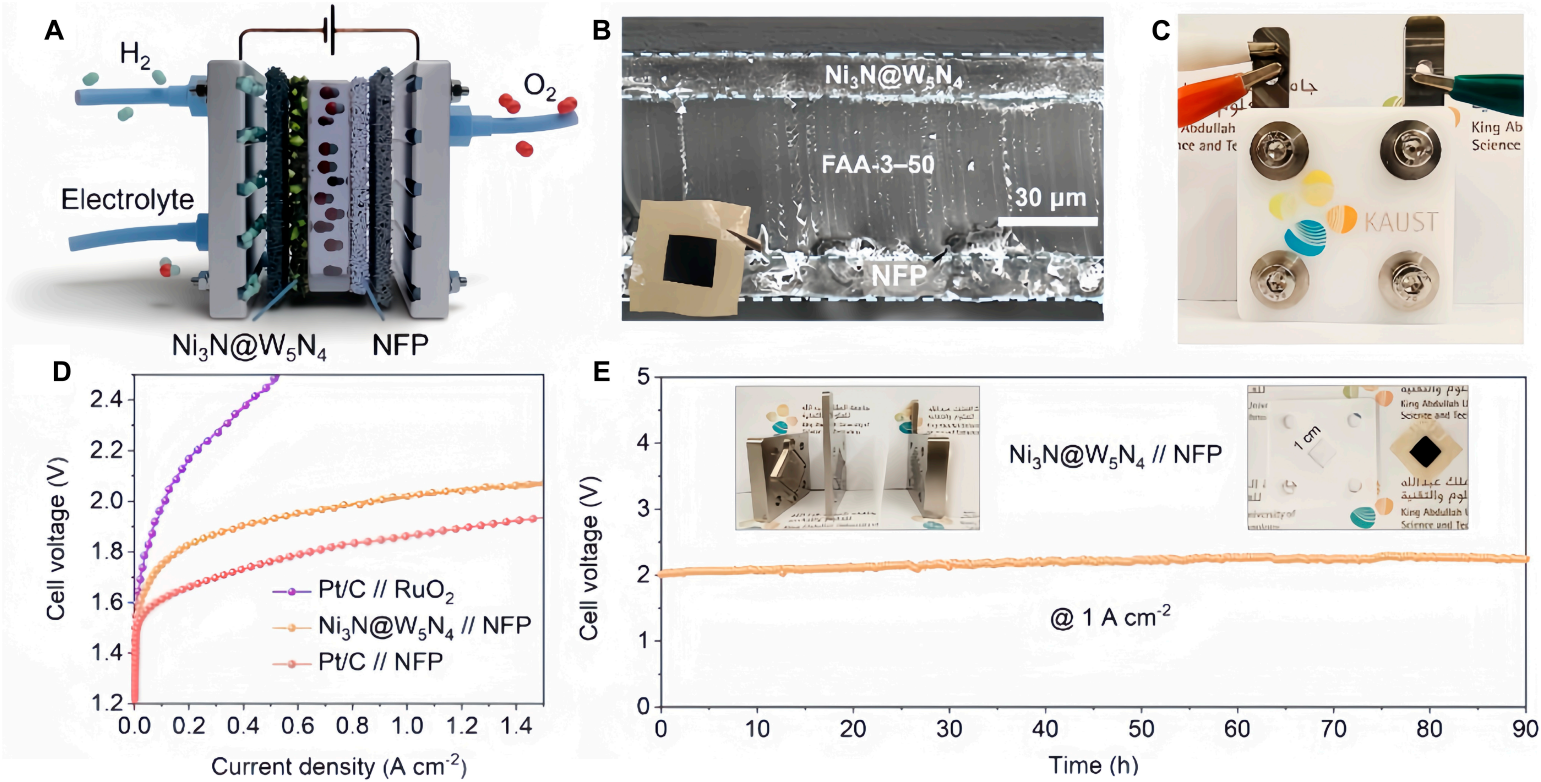
AEMWE device assembly and performance testing. (A) Schematic of the CCM in the AEMWE device. (B) Scanning electron microscope image of CCM. (C) Photo of the assembled flow cell. (D) Polarization curves of different CCMs (Pt/C//RuO_2_, Ni_3_N@W_5_N_4_//NFP, Pt/C//NFP). (E) Stability curve of Ni_3_N@W_5_N_4_//NFP CCM in flow cell at 1 A cm^−2^. Insets: Photographs of the main components of the flow cell [147].

Huang et al. [147] developed a heterogeneous structure consisting of ultrathin W_5_N_4_ shells and Ni_3_N nano-ions as an effective tethered singlet catalyst, where the built-in interfacial electric field creates biphasic metal nitrides with different lattice arrangements and figure of merit. Ni_3_N@W_5_N_4_ shows excellent properties in HER (60 mV@10 mA/cm^2^). When used as a cathode in AEMWE devices, the material exhibits stable performance at 1 A cm^−2^ for 90 h. The AEMWE flow cell assembled with a Ni_3_N@W_5_N_4_//NiFe phosphide (NFP) catalyst-coated membrane (CCM) demonstrated stable operation for 90 h at 1 A cm^−2^ without noticeable degradation in catalytic performance. In addition, Ni_3_N@W_5_N_4_//NFP achieved 1 A cm^−2^ at an applied voltage of 2.12 V (Fig. 15).

**Fig. 16.**
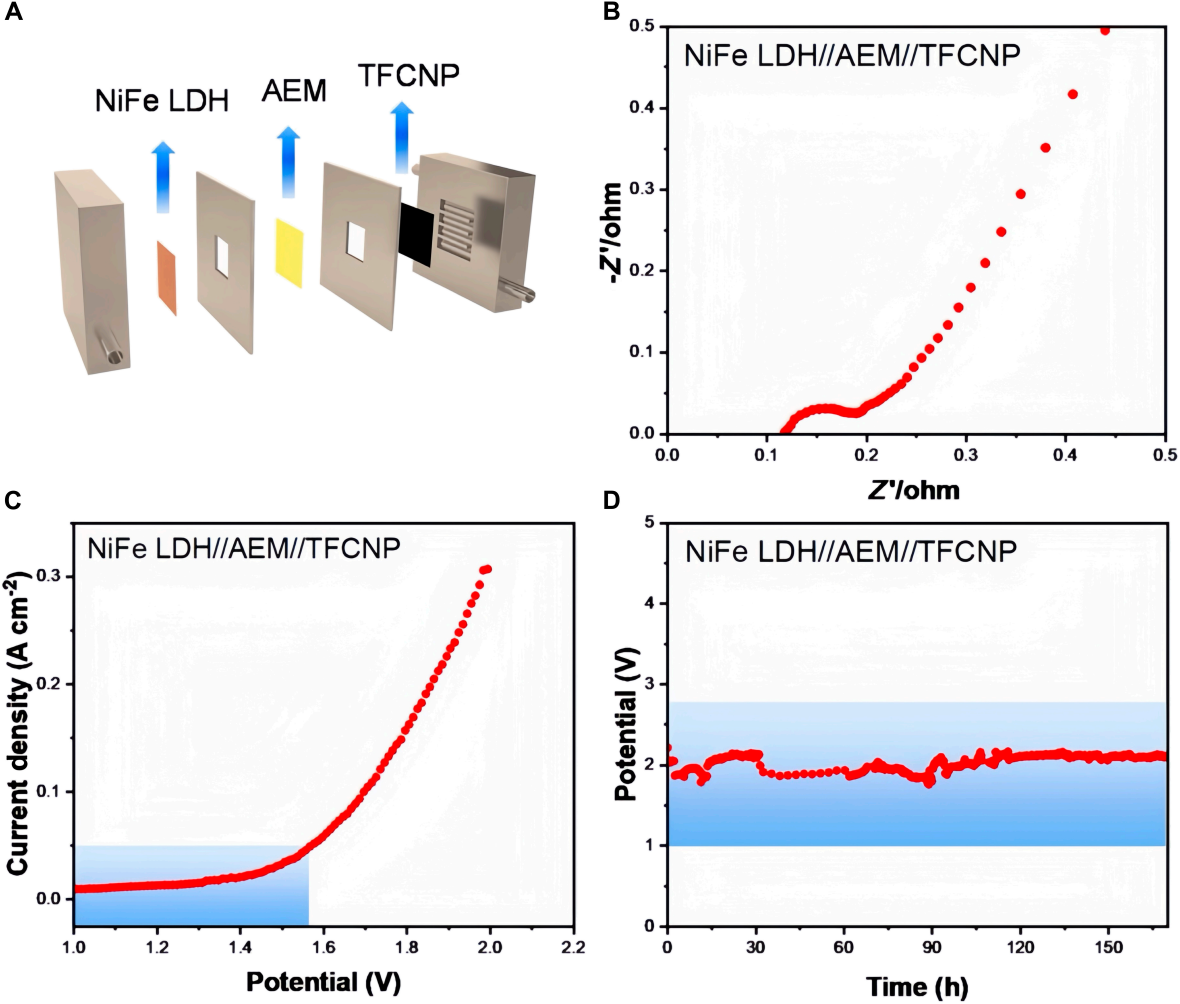
(A) Schematic of the AEM electrolyzer. (B) EIS diagram. (C) LSV polarization curves measured in NiFe LDH//AEM//TFCNP electrolyzer. (D) AEMWE performance test [148].

In 2024, Zhao et al. [148] developed a TiN nanowire-loaded FeCoNiP*_x_*-based catalyst (TFCNP), where TiN improved the charge transfer efficiency and increased the exposed active center. The TFCNP catalyst exhibited outstanding hydrogen reaction performance, with 72 mV at 10 mA cm^−2^. In addition, an AEMWE constructed with TFCNP as the cathode catalyst reached 50 mA cm^−2^ at a voltage of 1.57 V and operated stably under industrial operating conditions for more than 150 h, showing its potential for application in real-world water electrolysis for hydrogen production (Fig. 16).

##### MOF and POMs

**Fig. 17.**
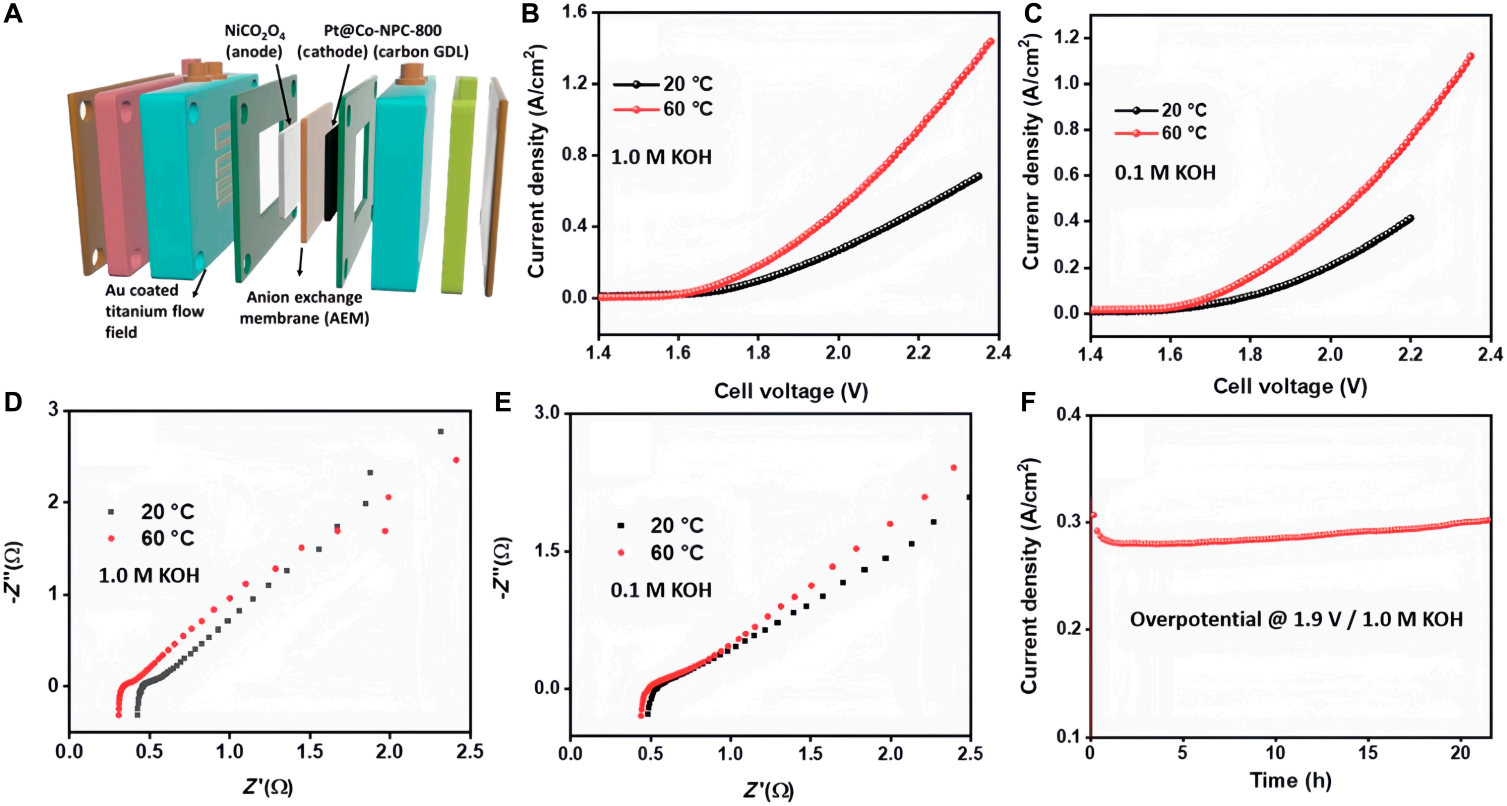
(A) Schematic of the AEMWE. (B) LSV plots. (C) LSV plots at 20 and 60 °C. (D) EIS plots for 1.0 M KOH. (E) EIS plots. (F) Stability measurements [152].

MOF materials have highly ordered porous structures and designable chemical compositions, which can be converted into metal oxides, carbon materials, or metal–carbon/nitrogen composites with abundant microporous structures and active sites by pyrolysis and etching, etc., and these derivatives exhibit good catalytic performance in HER reactions [114,149–151]. The researchers developed a carbon core–shell Pt nanoparticle/Co-MOF catalyst embedded in nitrogen-doped porous carbon derived from porphyrin Co-MOF by pyrolytically synthesizing Pt–aniline complexes at 600 to 800 °C. The catalyst was stabilized at 34 mV in alkaline electrolyte, reaching 10 mA cm^−2^. The catalysts remained stable for 96 h at 100 and 200 mA cm^−2^. The compounds have a high specific surface area, thus enhancing their HER activity. In addition, AEMWE using this catalyst achieved ~1.32 A cm^−2^ at 2.34 V battery. The Pt@Co-NPC catalyst was used in a variety of applications. This electrocatalyst offers a novel strategy for advancing high-performance AEMWE systems (Fig. 17) [152].

POMs, as a new type of HER materials, show great potential in AEM electrolysis for hydrogen production and are expected to become valuable hydrogen-exchange electrode materials. POMs have a unique molecular structure, and this structure endows them with a rich number of redox-active sites and adjustable electronic properties. In electrocatalytic hydrogen precipitation, POM can offer numerous active sites to promote the generation of hydrogen, thus improving the efficiency of the hydrogen precipitation reaction. In addition, POMs are low cost and easily functionalized, and can be further optimized for performance by modifying or compounding other materials [153–155].

For example, POM/Ni foam composites, prepared through a hydrothermal approach, demonstrate outstanding HER activity, achieving an overpotential of just 64 mV at 10 mA cm^−2^. Furthermore, the structure of POM can be precisely tuned by chemical synthesis methods to further optimize its catalytic properties. However, the poor electrical conductivity of POM and its tendency to fall off from the electrodes limit its application. To solve this problem, the researchers compounded paraformaldehyde with other conductive materials, thereby improving the conductivity of the electrodes [156]. In the future, with the in-depth study of the structure of POM, as well as the continuous progress of the composite material preparation technology, POM is anticipated to play a critical role in AEM electrolysis of water for hydrogen production, to provide strong support for the realization of efficient and green hydrogen production.

Future research directions for AEM’s water electrolysis technology will focus on exploring and optimizing nonprecious metal-based catalysts to reduce the reliance on expensive Pt group metals and lower the cost, as well as to improve the catalytic activity and stability through fine structure tuning and elemental doping; developing new nanostructures, multiphase catalysts, interface engineering and carrier materials, such as hierarchical structures, monatomic catalysts, 2D materials, and nano-cages to increase the active sites, promote mass transfer efficiency, improve catalytic efficiency, and reduce overpotential; and researching electrocatalysts suitable for a wide pH range and extreme conditions (high temperature and high pressure) while focusing on long-term catalytic efficiency and overpotential. We will also develop new nanostructures, multi-phase catalysts, interface engineering, and carrier materials such as hierarchical structures, monatomic catalysts, 2D materials, and nanocages to increase the number of active sites, promote the efficiency of mass transfer, improve the catalytic efficiency, and reduce the overpotential.

## Challenges and Future Directions for Large-Scale Application of AEMWE

### Long-term stability of AEM and electrode materials

#### Stability issues of AEM

The AEM serves as the central component of AEMWE, with its stability directly influencing the electrolysis efficiency and operational lifespan. However, the AEM faces several serious stability challenges, especially under long-term operating conditions, which have severely limited the commercial application of AEMWE. Traditional AEM often contains heteroatomic structures such as ether bonds and imine bonds, and these chemical bonds are easy to fracture under strong alkaline and high-temperature environments, leading to degradation of the membrane material. For example, some polymers are susceptible to electrochemical oxidation of their benzene rings and other groups under high current density and high voltage conditions, resulting in damage to the membrane structure and significant degradation of performance. In addition, the interfacial stability between the AEM and catalyst should not be neglected during long-term operation, especially under high current density and high gas generation rate conditions, where uneven gas release may lead to the stripping of the membrane from the catalyst. More seriously, under the synergistic effect of high current density, voltage, and temperature, the electrochemical stability of AEM faces great challenges, which could result in accelerated degradation of membrane materials and drastic performance degradation. These stability issues are intertwined and together constrain the long-term stable operation and commercialization of AEMWE [23,157].

To solve the above problems, a rigid twisted structure without impurity atoms can be introduced to construct the micropores, thus effectively avoiding the instability of impurity atoms (e.g., ether bonds and imine bonds) in the conventional microporous structure. This design not only improves the chemical stability of the membrane but also significantly reduces the intra-membrane transfer resistance and improves the ionic conductivity. In addition, the use of ionic membranes with low IEC improves the binding strength of the membrane, thereby extending its service life. High IEC ionic membranes with low water-absorption swelling can also be synthesized by introducing strategies such as multiple cations, polar interactions, and cross-linking. Finally, optimizing the structural design of membranes, such as using microporous structures, may improve the conductivity and stability of membranes [158].

#### Catalyst stability issues

Catalysts also face significant stability challenges during water electrolysis, especially under long-term operating conditions. First, during prolonged operation, catalyst particles may detach from the electrode surface, leading to performance degradation. Second, under OER conditions, the phenyl adsorbed in the ionic membrane is prone to electrochemical oxidation to generate compounds such as phenol, and these hinder the transport of reactants, thus reducing the activity of the catalyst. In addition, the stability problem of nonprecious metal catalysts is even more serious, for example, 3D TM-based catalysts (e.g., CoFe bimetallic Prussian blue analogs) tend to exhibit poor stability at industrial-grade current densities. Conventional nonprecious metal catalysts are prone to structural remodeling or dissolution at high current densities, resulting in a notable decline in catalytic efficiency [31,159].

To address the above problems, optimization can be carried out in the following aspects: First, the use of ionic membranes with low IEC values can enhance the adhesion of the catalysts to a certain extent, thus prolonging their service life. Second, by optimizing the catalyst preparation process, such as using encapsulation technology to confine the catalyst particles in a protective structure, it can effectively prevent them from falling off. In addition, selecting catalysts or polymer electrolytes with lower phenyl adsorption energy can alleviate the contamination of the catalyst surface by phenyl oxidation by-products. Finally, the application of encapsulation technology can significantly enhance the durability of catalysts [160].

### AEM electrolyzer electrolysis water hydrogen production operating costs and challenges

PEMWE has a first-mover advantage in the market, and related companies are gradually expanding their market share by improving hydrogen production efficiency and reducing costs. Over the past 5 years, the cost of PEM electrolyzer has dropped by 40%, and the trend of cost reduction of electric stack is still increasing. Therefore, the future development of AEMWE technology depends not only on its technological breakthrough but also on the competitive situation with PEMWE technology [161]. The following strategies can be used to reduce the manufacturing and cost of AEM electrolyzers: (a) Expanding the production scale can significantly reduce the unit cost. By realizing large-scale production, material procurement costs can be reduced and production efficiency can be improved. (b) Energy consumption and operating costs can be reduced by optimizing operating requirements, such as increasing current density and reducing electrolyte concentration. (c) Utilizing renewable energy sources for electrolysis can significantly reduce the cost of electricity. In addition, by optimizing the control strategy of the electrolyzer, the energy utilization efficiency of the system can be improved. (d) Enhance the stability of membrane materials and catalysts to extend the equipment’s service life. This not only reduces equipment replacement costs but also improves system reliability [162,163].

 In summary, although AEMWE technology offers significant advantages, many of which have already been realized or are potentially achievable from an industry perspective, the integration of these applications remains challenging. One of the key issues is the low ionic conductivity of AEM, which poses many challenges for large-scale application and commercialization. Solving these challenges requires in-depth research and innovation in core material development, catalyst stability improvement, system cost optimization, and competitive market strategies.

## Summary and Outlook

Green hydrogen, a promising renewable energy carrier, can be produced efficiently through AEMWE, an emerging pioneer in water electrolysis technology. This paper reviews the recent advancements in AEMWE technology, including electrocatalyst and device development. It begins by introducing the fundamental concepts and key parameters of AEMWE devices to provide a basic understanding. It then discusses several HER and OER electrocatalysts used as cathodes and anodes in AEMWE, highlighting how TM catalysts with superior electrocatalytic properties can enhance AEMWE. The paper further explores the long-term stability of anion-exchange membranes and catalysts, and the operational costs and challenges associated with industrial hydrogen production in AEM electrolyzers. Despite recent progress, AEMWE still faces some limitations in terms of efficiency and stability.

Drawing from the current research landscape, we identify several critical areas for future work: (a) enhancing the stability of AEMs, (b) developing more active HER and OER catalysts through innovative design strategies, and (c) evaluating the performance of AEMWE devices and optimizing their assembly. Overall, the progress in AEM development and electrocatalyst innovation highlights the promising potential of AEMWE. With in-depth research on the potential mechanism of AEMWE, we believe that this technology will continue to make breakthroughs and gradually expand its application areas.
